# Assessment Practice of Patient-Centered Outcomes in Surgical Neuro-Oncology: Survey-Based Recommendations for Clinical Routine

**DOI:** 10.3389/fonc.2021.702017

**Published:** 2021-08-11

**Authors:** Carolin Weiss Lucas, Mirjam Renovanz, Johanna Jost, Michael Sabel, Dorothee Wiewrodt, Marion Rapp

**Affiliations:** ^1^Center of Neurosurgery, Faculty of Medicine and University Hospital, University of Cologne, Cologne, Germany; ^2^Department of Neurology & Interdisciplinary Neuro-Oncology, University Hospital Tuebingen, Tuebingen, Germany; ^3^Department of Neurosurgery, University Hospital Tuebingen, Tuebingen, Germany; ^4^Department of Neurosurgery, Muenster University Hospital, Muenster, Germany; ^5^Department of Neurosurgery, Heinrich Heine University Hospital of Duesseldorf, Duesseldorf, Germany

**Keywords:** distress, burden, health-related quality of life (HRQL), patient-reported outcome (PRO), neurocognition, screening tools, brain tumor

## Abstract

The psycho-oncological burden related to the diagnosis of an intracranial tumor is often accompanied by neurocognitive deficits and changes in character, overall affecting health-related quality of life (HRQoL) and activities of daily living. Regular administration of adequate screening tools is crucial to ensure a timely detection of needs for support and/or specific interventions. Although efforts have been made to assure the quality of neuro-oncological care, clinical assessment practice of patient-reported outcomes (PROs) remains overall heterogeneous, calling for a concise recommendation tailored to neuro-oncological patients. Therefore, this survey, promoted by the German Society of Neurosurgery, was conducted to evaluate the *status quo* of health care resources and PRO/neurocognition assessment practices throughout departments of surgical neuro-oncology in Germany. 72/127 (57%) of registered departments participated in the study, including 83% of all university hospital units. A second aim was to shed light on the impact of quality assurance strategies (i.e., department certification as part of an integrative neuro-oncology cancer center; CNOC) on the assessment practice, controlled for interacting structural factors, i.e., university hospital status (UH) and caseload. Despite an overall good to excellent availability of relevant health care structures (psycho-oncologist: 90%, palliative care unit: 97%, neuropsychology: 75%), a small majority of departments practice patient-centered screenings (psycho-oncological burden: 64%, HRQoL: 76%, neurocognition: 58%), however, much less frequently outside the framework of clinical trials. In this context, CNOC affiliation, representing a specific health care quality assurance process, was associated with significantly stronger PRO assessment practices regarding psycho-oncological burden, independent of UH status (common odds ratio=5.0, p=0.03). Nevertheless, PRO/neurocognitive assessment practice was not consistent even across CNOC. The overall most commonly used PRO/neurocognitive assessment tools were the Distress Thermometer (for psycho-oncological burden; 64%), the EORTC QLQ-C30 combined with the EORTC QLQ-BN20 (for HRQoL; 52%) and the Mini-Mental Status Test (for neurocognition; 67%), followed by the Montreal Cognitive Assessment (MoCA; 33%). Accordingly, for routine clinical screening, the authors recommend the Distress Thermometer and the EORTC QLQ-C30 and QLQ-BN20, complemented by the MoCA as a comparatively sensitive yet basic neurocognitive test. This recommendation is intended to encourage more regular, adequate, and standardized routine assessments in neuro-oncological practice.

## Introduction

The diagnosis of an intracranial tumor confronts patients on the one hand with the burden of an oncological disease, but on the other hand also with neurocognitive deficits and changes in character, which overall affect health-related quality of life (HRQoL) and activities of daily living. Reliable patient-reported outcome measures (PROMs) can facilitate early recognition of psychosocial burden, depression, and anxiety and can lead to adequate support (1, 2). Accordingly, assessment and monitoring of neurocognitive function can play an important role in therapy and disease monitoring (3). Therefore, timely and closely followed patient-reported outcome (PRO) and performance-based assessments seem highly advisable to ensure a comprehensive neuro-oncological care, and have recently attracted increasing interest even beyond the context of clinical trials. However, to date there is no consensus regarding the best clinical and scientific practice of PRO and performance-based assessments in neuro-oncological patients.

Approximately 10 years ago, a standardized certification for neuro-oncology centers was implemented in Germany aiming at standardizing and improving patient care as comprehensively as possible. Since then, many positive developments have been observed in the field of neuro-oncology, driven by enhanced interdisciplinary cooperation. Despite all this, the sole requirement to date is to offer psycho-oncology counseling to at least 10% of brain tumor patients. Accordingly, clinical experience shows that the implemented standard operating procedures linked to certification have not yet reached a satisfactory level in terms of comprehensiveness and detail. For instance, adequate PROMs have not been included in official recommendations, and other important aspects, such as neurocognition, play a subordinate role, since no specifications are required. A fixed screening scheme to identify all types of related support needs would therefore be desirable as a standard operating procedure, even beyond the framework of certified neuro-oncology centers (CNOC; certified by the German Cancer Society [DKG]).

For this purpose and as a first step, we designed a survey, to describe the *status quo* of different assessment strategies applied throughout neuro-oncological units in CNOC and non-CNOC in Germany, also considering the university status as a potential confounder. Local organizational and health care structures are also considered to unravel interactions between existing structures as well as the clinical and scientific practice to evaluate psycho-oncological burden, HRQoL, and neurocognition in brain tumor patients. To conclude, this work provides a recommendation for a simple and little time-consuming assessment, based on the practical results of this survey and the literature.

## Material and Methods

### Study Design

The survey was designed by the authors on behalf of the neuro-oncological section of the German Society of Neurosurgery (DGNC) and was sent to all registered neurosurgical centers treating neuro-oncologic patients (i.e., n=127 centers) throughout Germany. The survey was conducted between November 2019 and April 2020. The heads of the neurosurgical departments or (if existing) of the specialized sub-units for neuro-oncological surgery were invited *via* electronic mail and/or telephone to participate in the survey. To ensure maximum survey response, multiple reminders were placed *via* electronic mail or phone calls to the departments’ secretaries. If no response was received after at least six reminders, the department was excluded from the study. The survey contained 28 multiple- and single-choice questions divided into four sections, mainly covering the following points (for detailed overview, cf. translated survey in the supplement): (i) center organization (CNOC, university hospital [UH], specialized neuro-oncologic outpatient clinic, caseload); (ii) health care structure (psycho-oncology, neuropsychology, palliative care); (iii) HRQoL assessment (practice and tools); (iv) assessment of psycho-oncological burden, depression, and anxiety (practice and tools); (v) assessment of neurocognition (practice and tools).

### Statistical Analysis

Data were stratified by institutional academic level (two levels: UH; others) and by affiliation to a CNOC (two levels: affiliated; not affiliated) to investigate the association of institution type and certification on the health care structure as well as on the practice of PRO assessments.

Differences between groups (stratified by, e.g., CNOC affiliation) with respect to binary outcomes such as the existence of health care structures were analyzed using the Mantel Haenszel Chi-squared test with continuity correction, controlling for the respective confounding co-factor (e.g., UH). In case the Mantel Haenszel test was significant, Fisher’s exact tests were calculated *post-hoc* for the respective subgroups.

For ordinal or continuous outcome variables, such as the time span between tumor diagnosis and first contact to palliative care, Wilcoxon’s rank sum test with continuity correction was calculated. Associations between ordinal or continuous variables and binary variables (e.g., caseload and UH) were analyzed using point-biserial correlations. To control for the interfering effect of a second significant factor, partial correlations were additionally calculated when appropriate. Statistically significant differences are generally reported as exact p-values. Whenever appropriate, a false discovery rate (FDR) correction (4) was applied (referred to as FDR-corrected throughout the manuscript). The statistical analysis was performed using R (version 3.6.3; R Studio version 1.1.463).

## Results

Out of 127 neurosurgical departments (including 36 UHs and 46 CNOCs), 72 departments (56.7%) participated. Four departments (3.1%) declined to participate; the remaining 51 departments did not respond despite being approached at least six times. 14 out of 16 German federal states returned the survey, with a certain overrepresentation of the districts North Rhine-Westphalia (21%) and Bavaria (15%).

### Center Organization

30 (42%) of the participating departments were part of UHs, as opposed to 37 (51%) university-affiliated teaching hospitals, and 5 (7%) district hospitals without university affiliation. 35 departments (49%) were part of CNOCs, and 60 departments (86%) declared to run a specialized neuro-oncologic outpatient clinic with a median caseload of 250 neuro-oncological consultations per year (range: 20-3000). This implies that this survey included 83% of all 36 German UHs running a neurosurgical unit and 76% of all 46 German CNOCs. Of note, there was a highly significant relationship between UH and CNOC status with most departments having the status of both (n=24/72) or neither UH nor CNOC (n=31/72; p<0.0001; **Table 1**).

**Table 1:** Squared table of department affiliation to CNOC *versus* UH.

Status	CNOC	No CNOC
University hospital	24	6
No university hospital	11	31

The chi-squared test shows a significant relationship between CNOC and UH status (p < 0.0001).

For this reason, the use of the Mantel-Haenszel test was considered appropriate (cf. Statistical analysis). An overview of the caseloads specifically referring to primary brain tumors (referred to as “caseload” throughout the manuscript) is provided in **Table 2**. In our sample, the caseload showed a strong, significant correlation with UH (r=0.63; p<0.0001) which remains significant when controlling for the factor CNOC affiliation using a partial correlation approach (r=0.55; p<0.0001). In contrast, the moderate correlation of the caseload with the CNOC affiliation of the department (r=0.37; p=0.002) did not survive when controlled for the UH status (r=0.04; p=0.55).

**Table 2:** Caseload and health care structure.

		Percentage					Statistical significance of stratifying factors	
		Overall	CNOC		No CNOC		Factor CNOC	Factor UH
			UH (n=24)	No UH (n=6)	UH (n=11)	No UH (n=31)	(controlled for factor UH)	(controlled for factor CNOC)
Primary brain tumor consultations per year*								
	<100	**26%**	4%	30%	0%	52%	r=0.04; p=0.72	r=0.55; p<0.0001
	100-199	**39%**	38%	60%	33%	38%		
	200-299	**17%**	25%	10%	33%	10%		
	≥300	**14%**	33%	0%	33%	0%		
	*[Reply rate]*	***[96%]***	*[100%]*	*[91%]*	*[100%]*	*[94%]*		
Health Care Structure**								
	Psycho-oncology *[Reply rate]*	**90%** ***[99%]***	100% *[100%]*	100% *[100%]*	100% *[100%]*	77% *[97%]*	X²=1.63; p=.20; cOR=na	X²=0.55; p=.46; cOR=na
	Palliative Care Inpatient *[Reply rate]*	**97%** ***[94%]***	96% *[100%]*	100% *[91%]*	100% *[100%]*	96% *[90%]*	X²=0.01; p=.92; cOR=1.3 [0.02;79.0]	X²=0.04; p=.84; cOR=0.6 [0.01;36.6]
	Outpatient *[Reply rate]*	**57%** ***[94%]***	71% *[100%]*	60% *[91%]*	67% *[100%]*	43% *[90%]*	X²=0.32; p=.57; cOR=1.7 [0.5;5.3]	X²=0.73; p=.39; cOR=2.0 [0.5;5.3]
	Neuropsychology *[Reply rate]*	**75%** ***[94%]***	92% *[100%]*	80% *[91%]*	100% *[100%]*	55% *[94%]*	X²=0.48; p=.49 cOR=2.29 [0.51;10.21]	X²=2.79; p=.095; cOR=6.81 [1.02;45.59]

Significant differences regarding health care structures are highlighted (statistical trends in light blue). Statistical tests: *partial Pearson correlations; **Cochrane Mantel-Haenszel test. Percentages are also provided by subgroups, i.e., UHs (as opposed to non-university institutions) and CNOCs. Overall percentages [reply rates] are highlighted in bold.

Of interest, 28 (47%) of the participating departments replied to perform awake neurosurgery on a regular basis, ranging from 32% without to 62% with CNOC affiliation, irrespective of the UH status (Cochrane Mantel-Haenszel test: cOR=3.5, p=0.002) and caseload.

### Health Care Structure

Except for departments without UH status nor CNOC affiliation (77%), psycho-oncology services were fully available in all other participating centers (**Table 2**), as reflected by a moderate, significant correlation of this care structure with caseload size (r=0.26; p=0.03). Across all centers, the availability of psycho-oncological support was higher for inpatients compared to outpatients (i.e., 78% *versus* 54%, respectively) and was mostly provided by psychologists (79%) and/or by medical staff (27%), and very rarely by pastors (1%).

Accordingly, inpatient palliative care was available in nearly all departments (97% overall), whereas the existence of outpatient palliative care services ranged from 43% to 71% (**Table 2**). In most centers, the respective services were provided by the hospitals and relatively rarely in collaboration with other institutions (psycho-oncology: 7%; palliative care: 15%, overall). The median time span between tumor diagnosis and first contact with palliative care was 39 weeks (i.e., 9 months), ranging from 1 to 87 weeks (i.e., 20 months), statistically unrelated to CNOC or UH status.

**Figure 1:**
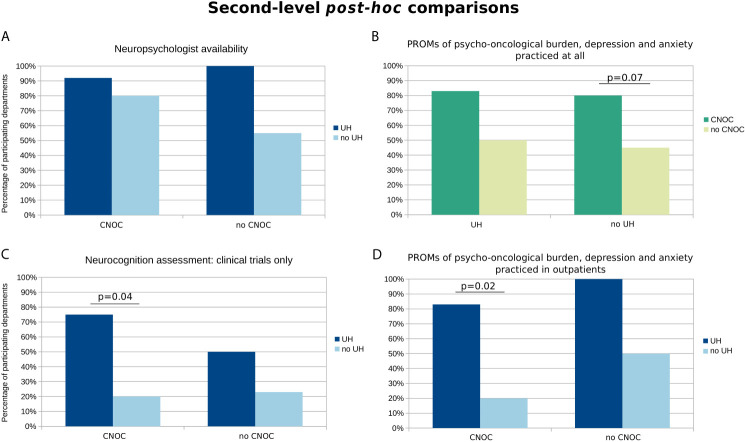
Second-level *post-hoc* comparisons of available health care structures and assessment practices influenced by quality assurance and institutional factors (i.e., either CNOC or UH affiliation as grouping factors). The y-axis represents the percentages of departments with **(A)** availability of the respective health care structures or **(B–D)** practice regarding the specific assessments. The selection of charts is based on statistically relevant group differences, at least on the level of a statistical trend (p < 0.1) according to the Cochrane Mantel-Haenszel test (cf. colored fields in **Tables 2** and **3**). Exact p-values according to post-hoc Fisher’s Exact tests (FDR-corrected) are provided if p < 0.1.

Overall, neuropsychological units/services existed in 75% of participating hospitals, ranging from 55% to 100% depending on department affiliation: there was a statistical trend towards better availability of neuropsychologists in UH (p=0.095) whereas the CNOC affiliation factor had no significant influence (**Table 2** and **Figure 1**). Accordingly, an utmost weak correlation was observed between neuropsychologist availability and caseload (r=0.21; p=0.09).

### Assessment of Psycho-Oncological Burden, Depression, and Anxiety

**Figure 2:**
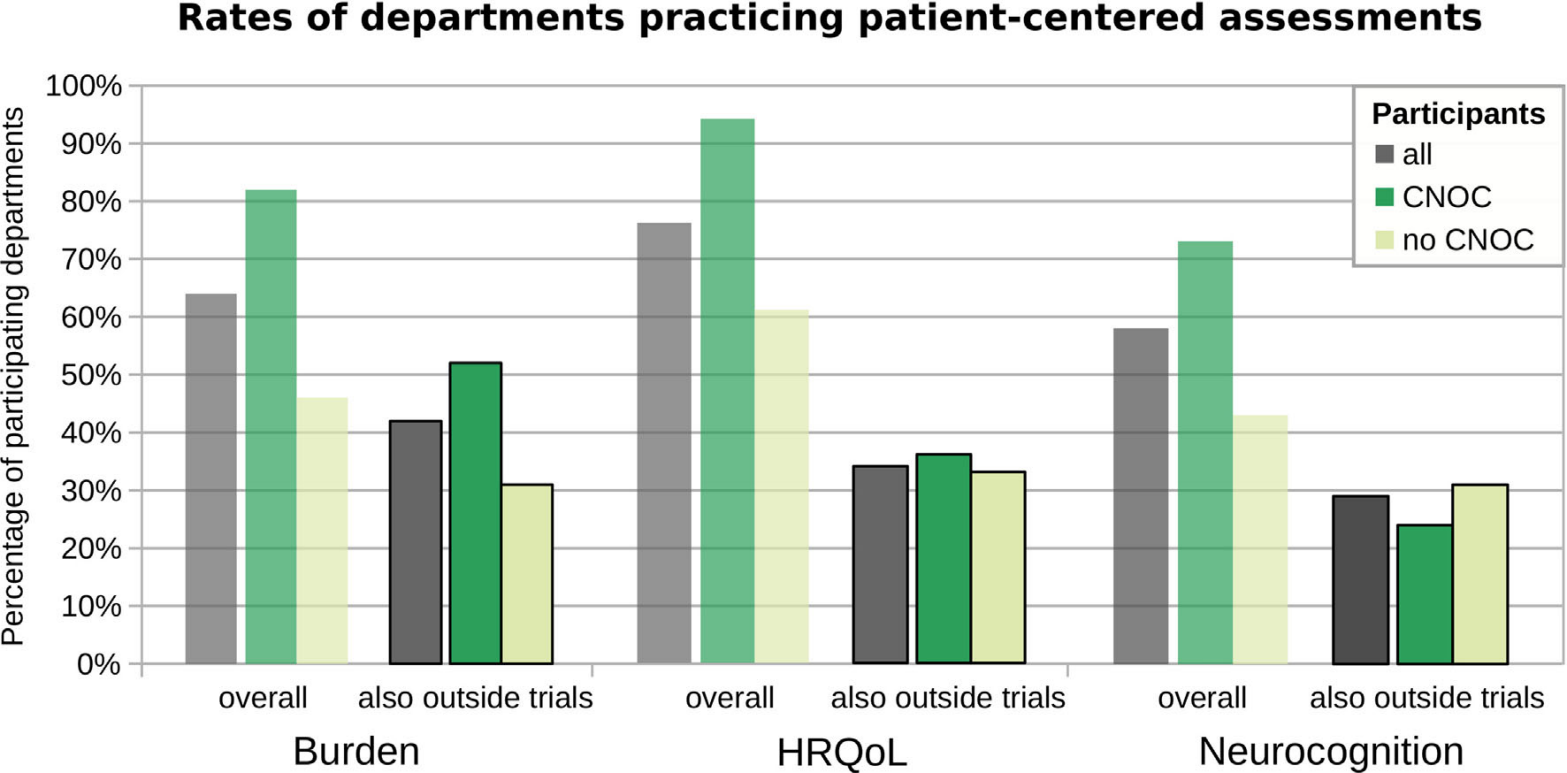
Assessment practice overall *versus* not exclusive to clinical trials. The y-axis represents the percentages of departments performing the respective assessment types, across all participating departments (grey) and grouped by CNOC (green) *versus* no CNOC (light green) status. Burden: psycho-oncological burden, depression, and anxiety.

The assessment of psycho-oncological burden, depression, and anxiety is practiced in most of the participating departments (i.e., 64%), more commonly across CNOCs (p=0.03; **Table 3**), but independent of the caseload (r=0.17; p=0.16). However, this influence of CNOC affiliation on assessment practice was not significant after correcting for exclusively study-related practice, i.e., when considering only assessments outside the context of clinical trials (**Figure 2**). Overall, the majority of patients (median estimation 80%) is assessed in departments which reported to perform PROMs of psycho-oncological distress, depression, and anxiety. Relatively rarely (20%), the caregivers were included in distress assessments, irrespective of department affiliation and certification (**Table 3**); however, significantly associated with higher caseloads (r=0.46; p=0.02).

**Table 3:** Regularity and indications of PRO and neurocognitive assessments.

		Percentage					Cochrane Mantel-Haenszel test	
		Overall	CNOC		No CNOC		Factor CNOC	Factor UH
			UH (n=24)	No UH (n=6)	UH (n=11)	No UH (n=31)	(controlled for factor UH)	(controlled for factor CNOC)
**Psycho-oncological burden, depression, and anxiety**								
	Assessments practiced *[Reply rate]*	**64%** ***[96%]***	83% *[100%]*	80% *[91%]*	50% *[100%]*	45% *[94%]*	X²=4.96; p=.03; cOR=5.0[1.4;18.0]	X²=0.10; p=.75; cOR=1.2 [0.3;4.5]
	Clinical trials only *[Relative reply rate]*	**34%** ***[100%]***	45% *[100%]*	13% *[100%]*	100% *[100%]*	15% *[100%]*	X²=0.10; p=0.75; cOR=1.2 [0.3;4.5]	X²=6.09; p=0.14; cOR=11.0 [1.5;82.2]
	Specific entities only *[Relative reply rate]*	**7%** ***[100%]***	0% *[100%]*	0% *[100%]*	0% *[100%]*	23% *[100%]*	X²=0.01; p=0.92; cOR=0 [nan;nan]	X²=0.65; p=0.42; cOR=0 [nan;nan]
	Caregiver included *[Relative reply rate]*	**20%** ***[100%]***	30% *[100%]*	0% *[100%]*	0% *[100%]*	23% *[100%]*	X²=0.11; p=0.74; cOR=0.7 [0.1;5.1]	X²=0.30; p=0.58; cOR=3.0 [0.4;23.9]
	Inpatients* [Relative reply rate]*	**100%** *[68%]*	100% *[75%]*	100% *[45%]*	100% *[17%]*	100% *[19%]*	nan	nan
	Outpatients *[Relative reply rate]*	**76%** ***[68%]***	83% *[75%]*	20% *[45%]*	100% *[17%]*	50% *[19%]*	X²=0.27; p=0.60; cOR=0.2 [0.01;3.3]	X²=5.21; p=0.02; cOR=23.3; [1.8;308.3]
**HRQoL**								
	Assessments practiced *[Reply rate]*	**76%** ***[97%]***	100% *[100%]*	70% *[91%]*	50% *[100%]*	63% *[97%]*	X²=2.63; p=0.11; cOR=3.0 [0.8;11.0]	X²=0.50; p=0.48; cOR=1.9 [0.5;7.4]
	Clinical trials only *[Relative reply rate]*	**55%** ***[100%]***	63% *[100%]*	57% *[100%]*	67% *[100%]*	42% *[100%]*	X²=0.01; p=0.91; cOR=1.4 [0.3;5.8]	X²=0.09; p=0.76; cOR=1.6 [0.4;6.5]
	Specific entities only *[Relative reply rate]*	**11%** ***[100%]***	21% *[100%]*	0% *[100%]*	33% *[100%]*	0% *[100%]*	X²=0.23; p=0.63; cOR=0.5 [0.04;7.0]	X²=1.12; p=0.29; cOR=inf. [nan;nan]
	Inpatients *[Relative reply rate]*	**93%** ***[55%]***	95% *[83%]*	100% *[14%]*	100% *[67%]*	83% *[32%]*	X²=0.01; p=0.91; cOR=1.6 [0.004;509]	X²=0.18; p=0.67; cOR=5.3 [0.01;2680]
	Outpatients *[Relative reply rate]*	**76%** ***[55%]***	80% *[83%]*	0% *[14%]*	100% *[67%]*	67% *[32%]*	X²=0.46; p=0.55; cOR=0 [nan;nan]	X²=1.2; p=0.3; cOR=inf. [nan;nan]
**Neurocognition**								
	Assessments practiced *[Reply rate]*	**58%** ***[96%]***	83% *[100%]*	50% *[91%]*	33% *[100%]*	45% *[94%]*	X²=1.82; p=0.18; cOR=2.4 [0.8;7.5]	X²=0.62; p=0.43; cOR=1.9 [0.6;5.8]
	Clinical trials only *[Relative reply rate]*	**50%** ***[100%]***	75% *[100%]*	20% *[100%]*	50% *[100%]*	23% *[100%]*	X²=0.1; p=0.7; cOR=1.4 [0.2;9.0]	X²=3.55; p=0.06; cOR=7.7 [1.2;47.6]
	Specific entities only *[Relative reply rate]*	**15%** ***[100%]***	15% *[100%]*	20% *[100%]*	0% *[100%]*	15% *[100%]*	X²=0.26; p=0.61; cOR=2.0 [0.2;23.4]	X²=0.38; p=0.60; cOR=0.5 [0.05;5.2]

Percentages of positive responses are provided by subgroups, i.e., UH and/or CNOC affiliation, along with the rate of replies to each question of the questionnaire (reply rate). Differences between groups (controlled for the alternative factor) are described according to Cochrane Mantel-Haenszel statistics. Results showing a statistical trend or significant association are highlighted (green: p < 0.05; blue: p < 0.1). In such cases, additional post-hoc tests (Fisher’s exact tests) were calculated for the respective subgroups (cf. **Figure 1**). COR, common odds ratio (95 percent confidence intervals of true common odds ratios provided in brackets). Nan, not computable. Overall percentages [reply rates] are highlighted in bold.

**Figure 3:**
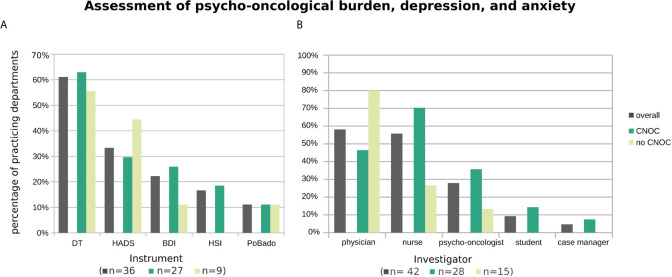
Applied psycho-oncological assessment instruments **(A)** and administering professionals **(B)**, overall and stratified by certification type of participating departments. Histograms are based on replies from *n* departments practicing assessment of psycho-oncological burden, depression, and anxiety (with *n* provided by subgroup in the x-axis label). Multiple instruments (or multiple types of professionals) were named by 39% (29%) of departments.

The most common PROM carried out to assess psycho-oncological burden, depression, and/or anxiety was by far the Distress Thermometer (DT; (5) 61% overall), followed by the Hospital Anxiety and Depression Scale (HADS; (6) 33% overall), the Beck Depression Inventory (BDI; (7, 8) 22% overall), and the Hornheider Screening Instrument (HIS; (9) 17% overall). The Basic Documentation for Psycho-Oncology [PO-Bado; (10)], which is an external assessment instrument, was used by four departments (i.e., 11% overall) in addition to at least one of the aforementioned PROMs (**Figure 3A**). When considering only the 22 departments which perform distress assessments (also) outside the context of clinical trials, the three most frequently used tools were the DT (64%), the HSI (23%), and the HADS (18%), followed by the BDI (14%) and the PO-Bado (9%).

Overall, the assessments were mostly performed by physicians (58%), followed by nurses (56%) and psycho-oncologists (28%), and rarely by students (9%) and case managers (5%) (**Figure 3B**). To account for the association between CNOC affiliation and assessment practice (in contrast to an utmost minimal association with UH), the descriptive data shown in **Figure 3** are stratified by CNOC.

### Health-Related Quality of Life Assessment

Although HRQoL assessment is practiced in the vast majority of departments (76%; **Table 2**), irrespective of their caseloads (r=0.18; p=0.15), the assessment is widely limited to clinical trials. Consequently, the percentage of centers with clinical routine practice in HRQoL assessment outside the context of studies reaches only 34%, statistically independent of their affiliations and caseload (**Figure 2**). Moreover, screening of brain tumor patients for HRQoL is generally irregular, even in departments that perform such screening (median 50% of patients, overall; see supplemental **Table S1**).

**Figure 4:**
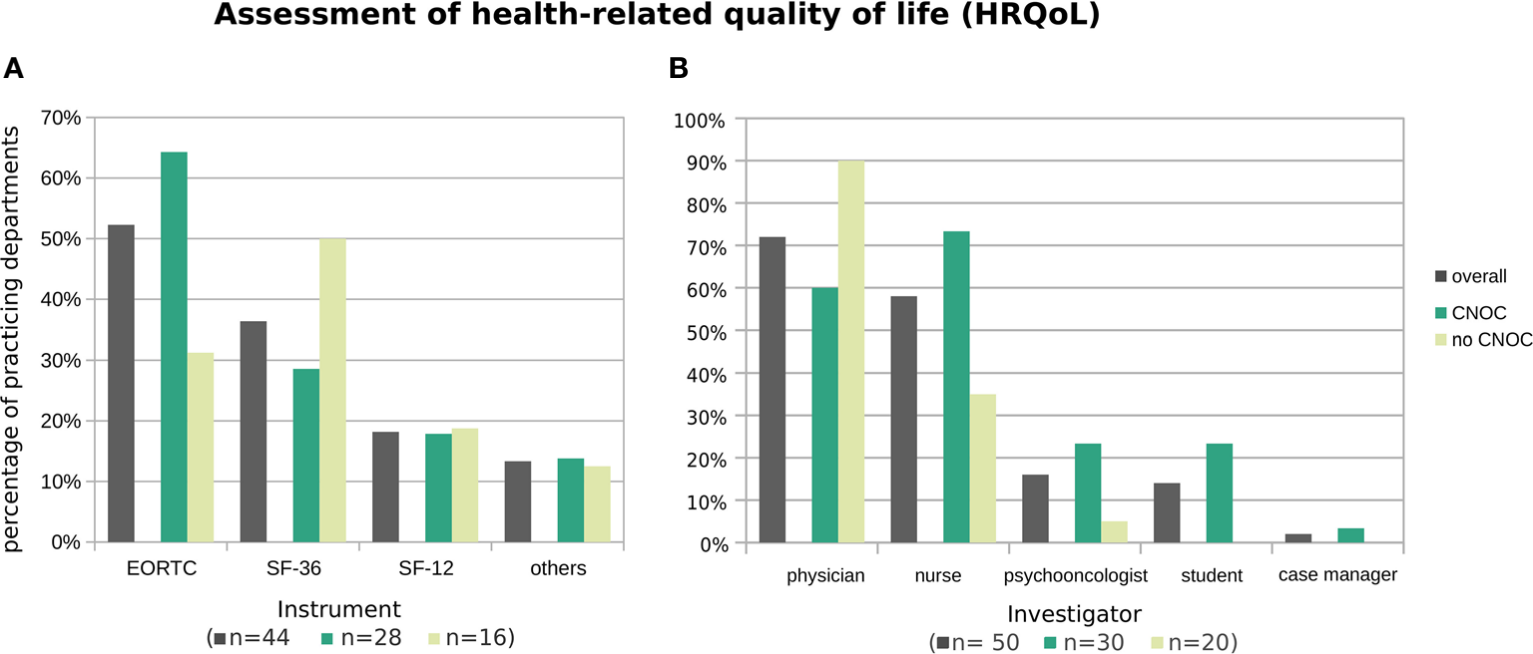
Practiced HRQoL assessment instruments **(A)** and administering professionals **(B)**, overall and stratified by CNOC affiliation. Histograms are based on replies from *n* departments practicing assessment of HRQoL (with *n* provided by subgroup in the x-axis label). Multiple instruments (or multiple types of professionals) were named by 27% (30%) of departments. Other instruments (all named once) were the Functional Assessment of Cancer Therapy [FACT; (11)], the 5-level EQ-5D version [EQ-5D-5L; (12)], the Hornheider Screening Instrument (HIS; (9) cf. Assessment of psycho-oncological burden, depression, and anxiety), the Barthel Index (13), the Aachen Life Quality Inventory [ALQI; (14)], and an unspecified instrument developed by the respective department.

**Figure 4A** shows that the most commonly used HRQoL screening instrument was the 30-items quality of life questionnaire of the European Organisation for Research and Treatment of Cancer (EORTC QLQ-C30) accompanied with its brain module (EORTC QLQ-BN20) (15), overall (52%) as well as in CNOC (64% *vs.* 31% no CNOCs). In contrast, outside CNOC departments, the Short Form Health 36 [SF-36; (16)] was mostly used (50% *versus* 29% in CNOCs; 36% overall). The shortened version of the SF-36 [i.e., SF-12; (17)] represented the third most frequent HRQoL assessment tool (24% overall; CNOCs: 18%; no CNOCs: 19%); further instruments were named by single centers (cf. **Figure 4** legend).

Additional analysis of the subset of 24 departments performing HRQoL assessments other than in the context of clinical trials revealed a similar but more diversified pattern (EORTC: 42%; SF-36: 26%; SF-12: 5%; others: 11%).

In line with the distress assessments, the HRQoL self-reports are again mostly obtained by physicians, followed by nurses; in contrast, case managers are only very rarely involved (**Figure 4B**).

### Neurocognitive Assessment

The overall rate of departments practicing neurocognitive assessments was 58% (**Table 2**), irrespective of the caseload. However, only 29% of participating departments (also) perform cognitive assessments unrelated to studies (statistically independent of center affiliation, certification, and caseload; **Figure 2**). Accordingly, the overall median percentage of brain tumor patients undergoing cognitive assessment in each department was low, i.e., 25% (with estimates ranging from 5% to 100%; cf. **Supplementary Table S1**), indicating that few centers follow regular clinical practice in this regard.

**Figure 5:**
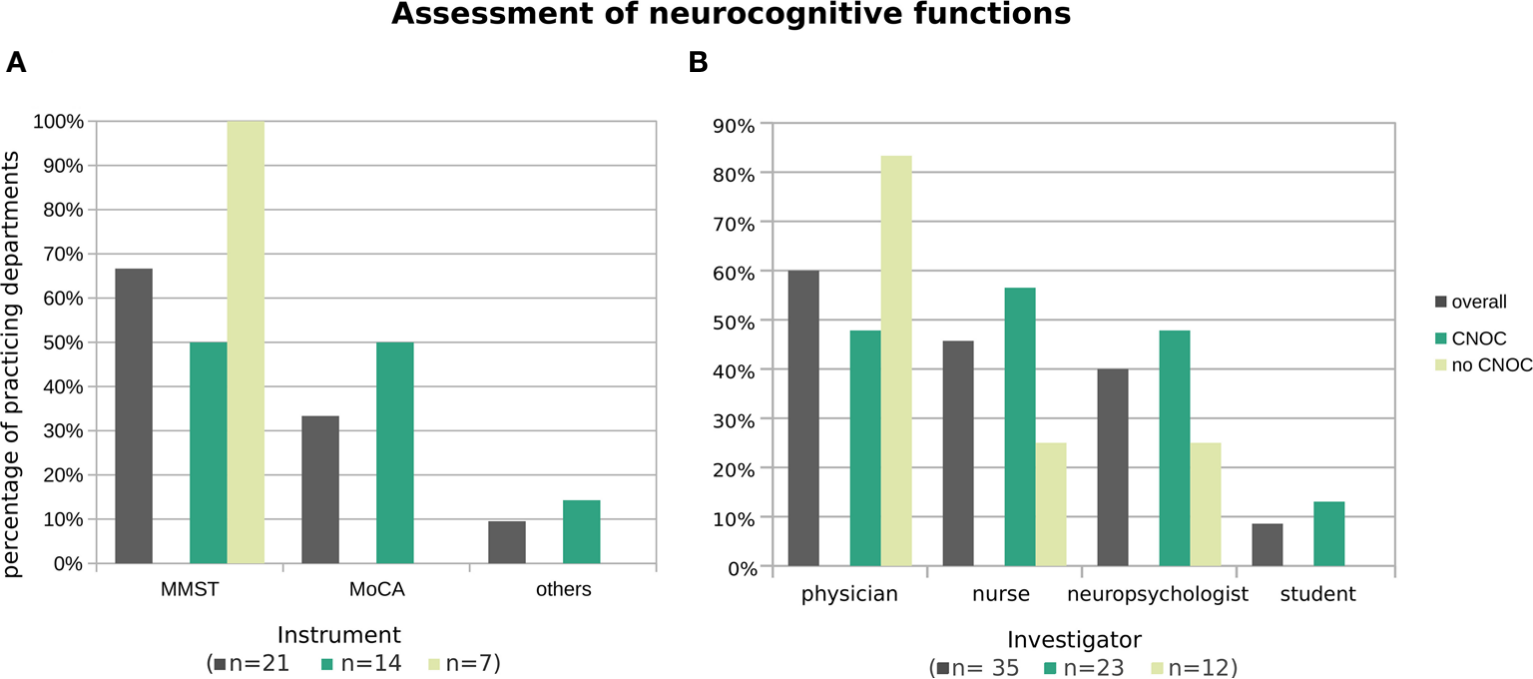
Practiced neurocognitive assessment instruments **(A)** and administering professionals **(B)**, overall and stratified by CNOC affiliation. Histograms are based on replies from *n* departments practicing assessment of neurocognitive functions (with *n* by subgroup in brackets in the x-axis label). Multiple instruments (or multiple types of professionals) were named by 10% (21%) of departments. Other instruments (each named once) were the dementia detection screening DemTect (19) and the screening battery of the NOA-19 study (20, 21), one naming of “others” was not further specified.

By far, the most commonly used screening instrument for neurocognitive functions was the mini mental status test (MMST) (18) (67% overall), particularly in centers without CNOC affiliation (**Figure 5A**). Accordingly, the MMST was practiced by seven out of nine (i.e., 78% of the) departments which practice neurocognitive assessment (also) beyond the exclusive context of clinical trials and answered this question.

The Montreal Cognitive Assessment [MoCA; (22)] represented the overall second most used neurocognitive test (33% overall) and reached the level of the MMST in CNOC (**Figure 5A**). When excluding the departments which practice neurocognitive assessments solely in clinical trials, the test was only named by one of the remaining nine centers.

Similar to the assessments of psycho-oncological burden, depression, and anxiety, as well as HRQoL, the tests were mostly performed by physicians. Nurses and neuropsychologists were also often included in the assessment, especially in CNOC (**Figure 5B**). Outside the context of clinical trials, the distribution was even between physicians and neuropsychologists (both 53%), followed by nurses (18%), whereas students played no role in collecting neurocognitive screening data (0%).

Regarding the time points of neurocognitive tests, the vast majority of the 35 centers practicing this assessment type investigate patients before (86%) and after (91%) surgery; 57% responded to conduct neurocognitive assessments during follow-up as well. Overall, 26% of the centers performing awake surgery include neurocognitive tests in the intraoperative setting (compared to 36% when considering only centers which practice neurocognitive screenings in clinical routine).

## Discussion

This survey investigated available health care structures and PRO as well as neurocognitive assessment practice in German neurosurgical departments, depending on their UH status and CNOC affiliation as well as on their caseloads of primary brain tumor patients. To the best of the authors’ knowledge, this is the first survey on this topic, providing a comprehensive overview due to the inclusion of the majority (57%) of the registered neurosurgical departments. Despite an overall good to excellent availability of relevant health care structures (i.e., referring to psycho-oncology, palliative care, and neuropsychology), the clinical routine assessment of relevant PROs, HRQoL assessment, and neurocognitive functions is limited, especially outside clinical trials. However, CNOC affiliation, representing a specific health care quality assurance process, was associated with significantly stronger PRO assessment practices regarding psycho-oncological burden, depression, and anxiety, independent of UH status.

### Assessment of Psycho-Oncological Burden and Adequate Support

Irrespective of tumor entity and prognosis, neuro-oncological patients are at risk for psychological comorbidities (23–25). A screening should be feasible within minutes and results have to be interpreted immediately in order to provide adequate support (26).

In line with our assumption from everyday clinical practice, psychosocial assessment was only carried out by a minority of participating departments outside clinical trials (42%). Although the majority of clinicians attach high importance to screening, its implementation in clinical routine is challenging (27), e.g., due to exhausted workload capacity of qualified staff. This applies particularly to screening instruments developed for cancer patients in general, which might be too complex and time-consuming for brain tumor patients, and thus difficult for them to manage. Even the application of seemingly quick and disease-specific self-report forms can bind significant staff resources, for instance, when patients need assistance in completing the form due to neurocognitive and/or other neurological deficits, or when the consecutive detection of needs requires further steps in patient management. On the other hand, the completion of screening forms by accompanying caregivers on behalf of patients reduces time expenditure for qualified medical personnel but leads to biased assessments. Compared to patients, it is even more difficult to address their relatives as well, reflected by only 20% of caregivers being included in distress assessments despite the generally heavy burden (28, 29). Although not yet widely used in clinical practice (and therefore not included in this survey), several established instruments are available to assess caregiver burden, e.g., the concise 12-items short form of the Zarit Burden Interview [ZBI] (30).

External factors such as certification requirements are leading to faster implementation of screening practice. This is underlined by the fact that psycho-oncological assessment was more common across CNOCs, where not only access to studies and specialized therapy is provided but also the required health care structures for patients are in place. However, implementing quality standards takes time. Looking at the past five years in the certification process, the numbers of initial psycho-oncological counselling have increased very slowly from 11.7% since 2015 (31) to 18.5% (32). Without the control of minimum requirements regarding the rate of patients to be psycho-oncologically assessed, the clinical assessment practice is prone to remain inconsistent.

The German psycho-oncology guideline recommends HADS-D, HSI, DT and PO-Bado, among others, as screening instruments. The BDI should not be regarded as a screening instrument for psycho-oncological distress/burden of disease. Nevertheless, it was indicated by 22% of participants and can be considered as a complementary and comparatively sensitive 21-items instrument to assess depression (as a disease to be medically treated with a considerable prevalence in brain tumor patients) (33). Since self-assessment might not be possible in every neuro-oncological patient, e.g., due to cognitive deficits, an external assessment by the physician can be helpful. If depression is suspected, this will be followed by a psycho-oncological consultation and specific diagnostics and, if necessary, therapy (cf. guideline unipolar depression of the Association of the Scientific Medical Societies in Germany [AWMF]) (34).

Regarding psycho-oncological screening instruments, the PO-Bado (11%) and the HSI (16%) were used rather rarely. The PO-Bado is an external assessment tool (10); hence, for the interpretation of its results, it should be considered that the physicians’ estimations do not necessarily reflect the patients’ perspectives (35). The HSI, a self-assessment screening instrument, as well as the PO-Bado, are widely used screening tools within Germany.

Both do not meet international quality criteria, which makes them rather unsuitable for international comparison.

The two most frequently used screening instruments were the HADS (33%) and the DT (61%). The HADS, an internationally well-established psycho-oncological screening instrument, refers exclusively to anxiety and depression, whereas the DT allows for the assessment of a much wider range of psychosocial problems and needs (36). Goebel and Mehdorn (37) validated the DT in brain tumor patients. Here, patients are considered to potentially carry a clinically relevant burden if the score is ≥ 6. In a previous work by Rapp et al. the relationship between HADS and DT was analyzed in more than 470 patients (26) resulting in the recommendation to consult a psycho-oncologist if the DT score is ≥ 5 and emotional problems ≥ 2.

The predominant use of the DT – at least in surgical neuro-oncology in Germany – might not only mirror the broad international acceptance of its short answer option but be also due to its simple, easily administrable, and non-stigmatizing character. The importance of these characteristics should not be underestimated in respect of the considerable prevalence of cognitive deficits in the target population, which interferes with the completion of long and complex questionnaires (38, 39). To address the specific needs of brain tumor patients, Goebel et al. have recently developed an adapted version of the problem list of the DT [HEAT; (38)], focusing on a more disease-specific needs assessment. This test still needs to be validated but could become a highly valuable PROM for brain tumor patients in the future (cf. **Table 4**).

**Table 4:** Recommendations for a basic, comprehensive assessment for brain tumor patients.

Assessment type/topic	Basic (available)	Perspectives *[/complementary]*
Psycho-oncological burden	DT	Targeted assessment for neuro-oncological patients [based on (37)]
HRQoL	EORTC-QLQ-C30, BN20	EORTC: Update BN-20 (40), *[EORTC item library* (41)*], [EORTC CAT* (42, 43)*]*
Neurocognition	MoCA	NOA-19 battery for glioblastoma (20, 21)

Along with our current recommendation, perspectives on promising future instruments are provided.

Currently, we recommend the DT as a psycho-oncological screening tool for brain tumor patients (**Table 4**).

### Health-Related Quality of Life Assessment

Serving as an independent predictor of therapy compliance and survival (44), HRQoL was the first PROM serving to evaluate new schemes of neuro-oncological therapy (45). In recent decades, its assessment has gained importance as an outcome measure of treatment response, far beyond the use as an endpoint in clinical trials (46, 47). In this regard, it is not surprising that this study showed a predominance of HRQoL assessment compared to other PROs evaluated (76% overall; up to 100% in CNOCs with UH status), although it also consumed relatively costly human resources (including 72% physicians and 58% nurses, overall). However, only 34% of participating departments practice HRQoL assessments outside clinical trials (independent of the institutional status). This demonstrates that the benefits of their use in improving clinical outcome prediction, complementing standard clinical outcomes, and detecting specific support needs are far from being exhausted. This finding might be influenced by (i) the lack of clear recommendations for HRQoL PROMs in current guidelines, (ii) the copyright protection of most common HRQoL PROMs making them less easily accessible, and (iii) logistic reasons related to the increased manpower required to ensure consequent assessment. In agreement with its predominance in European clinical trials, the EORTC QLQ-C30/BN-20 (15) was the most commonly used instrument (42%) in German centers, too. Notwithstanding its excellent quality in terms of internal consistency, content validity, and construct validity (48), as well as its validation for the specific group of neuro-oncological patients, this comparatively long 50-item questionnaire may be hard for patients to cope with, especially when being part of a comprehensive and repeated assessment based on multiple PROMs. This might be one reason why shorter HRQoL assessment tools (i.e., the 36-item SF-36 or its 12-item short form) (16, 17) were ranked second in frequency of use by the departments participating in this survey (SF-36: 26%; SF-12: 5%), especially by departments without CNOC affiliation, which are generally less influenced by specific requirements of clinical trials. Therefore, prospective studies and novel computerized concepts such as the computerized adaptive test version of the EORTC QLQ-C30 [EORTC CAT; (42, 43)] are highly appreciated. In the upcoming version, the authors intend to achieve a maximum PROM quality whilst reducing time needed to complete the questionnaires.

In summary, we presently recommend the EORTC QLQ-C30/BN-20 to assess quality of life in brain tumor patients (**Table 4**).

### Neurocognitive Assessment

The practice of neurocognitive assessment is rather limited (i.e., 58% overall; 29% outside clinical trials) and widely restricted to relatively simplistic dementia screening tools despite an apparently good overall availability of qualified investigators (e.g., almost 100% neuropsychologists, especially in UHs). Recent literature discussed brief cognitive screenings to be insensitive to important cognitive symptoms; thus, rendering them inadequate (49). The vast majority of departments assessing neurocognitive functions reported to use the well-known and easily administrable MMST, even more if outside the context of clinical studies. In contrast to its broad acceptance, the MMST, originally developed for dementia screening, demonstrates relatively low sensitivity regarding the detection of cognitive deficits in brain tumor patients (50). In comparison, another dementia screening test, i.e., the MoCA, which also allows for a relatively time-efficient and well standardized test administration, was reported to perform significantly better in neuro-oncological patients (p<0.0001) (51). This might explain why the MoCA is used relatively frequently in clinical trials, almost overtaking the MMST in departments with considerable study activity (i.e., CNOCs).

More sensitive but also more time-consuming and potentially burdensome, neurocognitive test batteries are apparently very rarely part of clinical assessments in German neurosurgical departments (2 centers, 3% overall), although nowadays generally recommended (52). This is noteworthy since cognitive deficits correlate strongly not only with HRQoL and activities of daily living (53) but also with tumor progression (54) and survival (55). Accordingly, timely detection of neurocognitive deficits using appropriate, sensitive screening instruments appears advisable to enable the responsible physicians to recommend adequate diagnostic, supportive, or therapeutic interventions. Here, a comprehensive but shortened neurocognitive testing instrument could help to improve assessment practice, and thus the detection of cognitive deficits and related support needs. It would need to be tailored to the limited attention span and coping ability of (newly diagnosed) neuro-oncologic patients and sufficiently standardized to be administered by trained nurses or students. Such a neurocognitive test battery including five parallel versions is currently being evaluated for use in glioblastoma patients in the multicentric NOA-19 study (20, 21). The results will help to find an appropriate neurocognitive test strategy for brain tumor patients in the future.

For now, we recommend the MoCA as basic assessment tool for neurocognition (**Table 4**).

### Impact of Quality Assurance Strategies and Personnel Structure

The higher rate of psycho-oncological PRO assessments in the subgroup of CNOC departments demonstrates the potential of quality-assuring instruments to make a change in assessment practice, building the ground for the detection of support needs and subsequent initialization of supporting interventions. Therefore, the integration of further patient-centered outcome assessments into quality assurance strategies (such as standard-operating procedures, guidelines, or controlled certification requirements) seems advisable to achieve optimized health care standards in neuro-oncology also outside clinical studies. In this context, concrete recommendations regarding an ideal time frame for first contact to palliative care units might also be valuable, as early and regular contact with a palliative care team beginning within a few weeks after first tumor diagnosis has been shown to improve HRQoL, symptom burden, and mood in patients of other oncological entities (56, 57). Moreover, advanced care planning (ACP) is clearly appreciated by the vast majority of neuro-oncological patients and is highly dependent on the patient’s general, psychological, and neurocognitive state still being adequate (58, 59). These and other points favoring an integrative palliative care approach argue for earlier involvement of palliative care teams than currently practiced in German departments of surgical neuro-oncology (with a median of 9 months, up to 20 months in our data set).

Another key finding of this work is the imbalance between existing health care structures and available instruments for assessing clinically clearly important PROs and neurocognition on the one hand, and the heterogeneous and incomplete clinical practice of such assessments on the other. As mentioned earlier, one reason for this might be the traditional dependency of PROM and neurocognitive assessments on highly qualified, high-cost personnel like physicians. Especially, the completion of traditional PROMs, i.e., self-report forms to be completed by patients, could be undertaken by less costly staff if appropriate training was to be provided. To which extent this model is transferrable to neurocognitive testing depends on the degree of standardization and the ease of administration of the tests used (which in our view are sufficiently high regarding, e.g., the MMST or the MoCA test).

### Strengths and Limitations

This survey, addressing all registered centers of surgical neuro-oncology in Germany to avoid selection bias, draws a rather comprehensive picture of the neuro-oncological PRO practices in the country. Even pursuing this inclusive approach, we achieved an excellent participation rate of 57% (n=72/127, including n=42 non-UHs) compared to recent surveys in the field of neuro-oncology which followed a similar inclusion strategy [e.g., 5%; n=362/7280; (60)]. Other surveys reporting response rates in the range of 36% (61) up to 75% (62) come with the limitation of addressing a highly selected group of centers [e.g., 28 centers across eleven European nations; (62)].

Despite this methodological strength, the survey is not fully representative due to (i) the missing centers, especially regarding non-UHs and non-CNOCs (selection bias), as well as (ii) missing values due to incomplete surveys/responses, and (iii) the at least potential subjectiveness/rater dependency of several survey items (since the data are based on information provided by medical consultants rather than on official/reliable statistics of the respective institutions). Moreover, (iv) the survey was addressed to neurosurgical units as one representative part of integrative neuro-oncological care centers. To provide a more comprehensive overview of assessment practices dependent on the stages of treatment/disease (and on the distinct disciplines involved) was beyond the scope of this work and will be subject to an upcoming survey.

In the present inquiry, the existence of certain institutional and medical structures was surveyed (e.g., presence of a neuropsychologist), whereas the extent to which this (personnel) structure is actively involved in the assessment of brain tumor patients was not. Moreover, it should be emphasized that the existence of health care structures and practice of assessments (to detect neurocognitive and psycho-oncological needs) do not *per se* lead to improved quality of care – unless followed by adequate interpretation of the outcomes and timely initiation of appropriate measures. The question of which resources and assessment tools, mediated by consecutive interventions/support, have a significant impact on health care quality was beyond the scope of this work and might be further addressed in a prospective study.

## Concluding Recommendations

The *status quo* of PRO and neurocognition assessment in surgical neuro-oncology shows that despite existing care structures, even in CNOCs there are no consistent standard procedures. As a consequence, many patients and caregivers are left alone with their needs and burdens. Widespread adoption of screening tools is essential to implement regular PRO and neurocognitive assessments in clinical practice. Therefore, screening tools are best suited when they bridge the gap between high test quality and practical considerations: tests should be as familiar as possible to the hospital staff, little time-consuming, and easy to perform and to evaluate. With regard to the results of this survey and literature, we hope that our concise recommendation (provided in **Table 4**) will encourage more regular, appropriate and standardized routine assessments in neuro-oncological practice.

## Data Availability Statement

The raw data supporting the conclusions of this article will be made available by the authors, without undue reservation.

## Author Contributions

CW: conception and design, acquisition, analysis and interpretation of data, manuscript drafting and revision. MRe, DW, and MRa: conception and design, acquisition and interpretation of data, manuscript drafting and revision. JJ: acquisition and interpretation of data, manuscript drafting and revision. MS: conception and design, manuscript revision. All authors contributed to the article and approved the submitted version.

## Funding

This work received financial support from the University of Cologne, Faculty of Medicine, to cover the publication costs.

## Conflict of Interest

The authors declare that the research was conducted in the absence of any commercial or financial relationships that could be construed as a potential conflict of interest.

## Publisher’s Note

All claims expressed in this article are solely those of the authors and do not necessarily represent those of their affiliated organizations, or those of the publisher, the editors and the reviewers. Any product that may be evaluated in this article, or claim that may be made by its manufacturer, is not guaranteed or endorsed by the publisher.
