# Supplementary material for: Assessment Practice of Patient-Centered Outcomes in Surgical Neuro-Oncology: Survey-Based Recommendations for Clinical Routine
Source: Front Oncol. 2021 Aug 11;11:702017. doi: 10.3389/fonc.2021.702017 (PMC8386174; doi:10.3389/fonc.2021.702017)
Supplement: Supplementary file 1 [file DataSheet_1.pdf]

Survey from the Neuro-Oncology Section of the DGNC

1. Clinical Structure:

Federal state:____________________

☐ University Clinic
☐ Teaching Hospital
☐ Hospital

☐ Neurosurgical Department
☐ Neurological Department
☐ Radiotherapy Department

Is a specialized neuro-oncological outpatient ambulance available?

☐ yes ☐ no

Brain tumor surgeries/year

☐ <100
☐ >100
☐ >200
☐ >300

Awake surgeries

☐ rather seldom
☐ regularly

Neuro-oncologic consultations or outpatient cases
of brain tumors/year (approx.): ____________________

For radiotherapists:
Initial radiation treatments (of brain tumors) per year (approx.): ____________________

Neuro-oncology center certified according to DKG criteria?

☐ yes ☐ no

Psycho-oncologist available?

☐ yes ☐ no if yes: ____________________ hrs/week

If yes: ☐ in own clinic ☐ in cooperation

Main profession of the psycho-oncologist?

☐ physician
☐ psychologist
☐ social worker
☐ healthcare chaplain
☐ other: ______________
☐ not sure / unknown

What is the organizational structure of psycho-oncology?

☐ inpatient ☐ outpatient ☐ both
☐ on-site ☐ consultation service
☐ just once ☐ longer-term therapy

If longer-term therapy: specific interventions? ______________

Palliative care available?

☐ outpatient ☐ inpatient ☐ both

If yes: ☐ in own clinic ☐ in cooperation

In your estimate, how many weeks/months after the initial diagnosis of GBM does the first contact with palliative care occur on average?
Weeks/months ____

Neuropsychologist available?

☐ yes ☐ no if yes: ____ hrs/week

If yes: ☐ in own clinic ☐ in cooperation

2. Assessment of health related quality of life in brain tumor patients:

On regularly base ☐ yes ☐ no

If yes :
☐ only in the context of clinical studies
☐ only for certain diagnoses ______________
☐ estimated in ____ % of patients
☐ inpatient only/ ☐ outpatient only / ☐ both

Is assessed by:

- ☐ nursing staff
- ☐ medical staff
- ☐ students
- ☐ study nurse
- ☐ case manager
- ☐ social worker
- ☐ psycho-oncologist

Which questionnaire:

- ☐ EORTC-C30 ☐ + BN20
- ☐ SF-36
- ☐ SF-12
- ☐ other: ____________________

3. Assessment of distress

On regularly base ☐ yes ☐ no

If yes :
- ☐ only in the context of clinical studies
- ☐ only for certain diagnoses: ____________________
- ☐ estimated in _____ % patients
- ☐ inpatient only/ ☐ outpatient only / ☐ both

Is assessed in:

- ☐ patients
- ☐ caregivers

Is assessed by:

- ☐ nursing staff
- ☐ medical staff
- ☐ students
- ☐ study nurse
- ☐ case manager
- ☐ social worker
- ☐ psycho-oncologist

Which questionnaire?

☐ Distress Thermometer
☐ HADS
☐ PO-Bado
☐ BDI (Beck’s Depression Inventory)
☐ other: ____________________

4. Assessment of neurocognition

On regularly base ☐ yes ☐ no

If yes : ☐ only in the context of clinical studies
☐ only for certain diagnoses: ____________________

☐ estimated in _____ % patients
☐ inpatient only/ ☐ outpatient only / ☐ both

preoperative	☐ yes	☐ no
postoperative	☐ yes	☐ no
intraoperative	☐ yes	☐ no
follow-up	☐ yes	☐ no
standardized protocol?	☐ yes	☐ no
NOA-19 protocol	☐ yes	☐ no

Which Screening:

☐ MMST ☐ MoCA
☐ other: ____

Is assessed by:

☐ nursing staff
☐ medical staff
☐ students
☐ study nurse
☐ case manager
☐ social worker
☐ psycho-oncologist
