# Supplementary material for: Assessment Practice of Patient-Centered Outcomes in Surgical Neuro-Oncology: Survey-Based Recommendations for Clinical Routine
Source: Front Oncol. 2021 Aug 11;11:702017. doi: 10.3389/fonc.2021.702017 (PMC8386174; doi:10.3389/fonc.2021.702017)
Supplement: Supplementary file 2 [file Table_1.pdf]

Table S1: Estimated percentage of all brain tumor patients (treated by the department) receiving regular assessments in either clinical or study context, overall and by department affiliation. Absolute and relative numbers of replies are provided (in italic letters) in relation to all departments included in the survey, next to estimated percentages of brain tumor patient assessments due to either clinical routine or study practice. Missing responses were handled as missing values (irrespective of whether the department performed such assessments at all). Please note that response rates to this question (provided in the table) were low, so results should be interpreted with caution.

Assessment type	Overall	UH vs. others	CNOC vs. others
**Psycho-oncological distress, depression and anxiety**			
N replies (% of total)	*17/72 (24%)*	*11/30 (37%) vs. 6/42 (14%)*	*13/35 (37%) vs. 4/37 (11%)*
Estimated percentage of patients assessed (median [range])	80% [5;100%]	70% [15;100%] vs. 85% [5;100%]	70% [5;100%] vs. 85% [20;90%]
**HRQoL**			
N replies (% of total)	*22/72 (31%)*	*13/30 (43%) vs. 9/42 (21%)*	*13/35 (37%) vs. 9/37 (24%)*
Estimated percentage of patients assessed	50% [10;100%]	40% [10;100%] vs. 90% [20;90%]	50% [10;100%] vs. 70% [20;90%]
**Neurocognition**			
N replies (% of total)	*16/72 (22%*	*7/30 (23%) vs. 9/42 (21%)*	*9/35 (26%) vs. 7/37 (19%)*
Estimated percentage of patients assessed	25% [5;100%]	20% [5;50%] vs. 50% [5;100%]	20% [5;80%] vs. 50% [5;100%]
